# Decoding Multifaceted Roles of Sleep-Related Genes as Molecular Bridges in Chronic Disease Pathogenesis

**DOI:** 10.3390/ijms26072872

**Published:** 2025-03-21

**Authors:** Wenyuan Wang, Linjie Zhao, Zhiheng He, Yang Zhao, Guijie Jiang, Chengjun Gong, Yan Zhang, Jiafeng Yu, Tingming Liang, Li Guo

**Affiliations:** 1 State Key Laboratory of Flexible Electronics (LoFE) & Institute of Advanced Materials (IAM), School of Chemistry and Life Sciences, Nanjing University of Posts & Telecommunications, Nanjing 210023, China; q22010105@njupt.edu.cn (W.W.); 1223014120@njupt.edu.cn (L.Z.); 1023173006@njupt.edu.cn (Z.H.); b22100623@njupt.edu.cn (Y.Z.); 1223014119@njupt.edu.cn (C.G.); b21080424@njupt.edu.cn (Y.Z.); 2 School of Life Science, Nanjing Normal University, Nanjing 210023, China; 241212073@njnu.edu.cn; 3 Shandong Provincial Key Laboratory of Biophysics, Institute of Biophysics, Dezhou University, Dezhou 253023, China; jfyu1979@126.com

**Keywords:** sleep, sleep-related gene, gene alteration, chronic diseases

## Abstract

Sleep is a fundamental process essential for all organisms. Sleep deprivation can lead to significant detrimental effects, contributing to various physiological disorders and elevating the risk of several diseases. Investigating the relationship between sleep and human diseases offers valuable insights into the molecular mechanisms governing sleep regulation, potentially guiding the development of more effective treatments for sleep disorders and associated diseases. This study explored the roles of sleep-related genes in biological processes and their associations with chronic diseases, mainly including neurological, metabolic, cardiovascular diseases, and cancer. Additionally, an analysis on the sleep-related genes was also performed to understand the potential role in tumorigenesis. This review aims to enhance the understanding of the link between sleep-related genes and chronic diseases, contributing to the development of novel therapeutic approaches targeting sleep and circadian rhythm-related chronic diseases.

## 1. Introduction

Sleep is a fundamental and vital behavior in most organisms, influencing various biological aspects, including cognitive function, emotion, and memory [1]. Humans exhibit a unique sleep pattern, consisting of two distinct phases: rapid eye movement (REM) sleep and non-rapid eye movement (NREM) sleep. According to established standards, NREM sleep is subdivided into 4 Stages, while REM sleep is characterized by rapid eye movements and heightened brain wave activity [2]. As individuals transition into Stage 1, consciousness gradually fades, with a decrease in α waves and the emergence of θ waves. Stage 2 sleep is marked by the appearance of K complexes, which consist of negative sharp waves followed by high-amplitude positive slow waves, and sleep spindles, characterized by 12–16 Hz oscillations. Stages 3 and 4 represent deep sleep, dominated by δ waves. In Stage 3, δ waves account for 20–50% of the time, whereas in Stage 4, they exceed 50%. At these stages, muscle activity decreases, body temperature and metabolic rate drop, and individuals become harder to awaken. REM sleep, distinguished by rapid eye movements, low muscle tone, and active brain waves, is typically associated with dreaming [2]. REM sleep is a critical phase of the sleep cycle, closely linked to memory consolidation and emotional regulation. NREM sleep is involved in energy conservation and neural recovery. Both stages are essential for maintaining physiological balance: NREM sleep facilitates recovery and REM sleep supports periodic brain activation, local restoration, and emotional regulation [1,3,4]. Chronic sleep and wake disturbances can lead to long-term health consequences. Various sleep disorders, such as delayed sleep onset, frequent awakenings, and daytime sleepiness, may signal suboptimal health conditions and even contribute to the onset of certain diseases. Sleep regulation mechanisms are complex, and there is growing interest in understanding the molecular pathways governing sleep and wakefulness. Recent advances in technology have identified numerous genes associated with sleep duration and sleep homeostasis maintenance, including those related to neural circuits such as ion channels (e.g., *Kcnn2*, *Kcnn3*), neuropeptides (e.g., orexin), and neurotransmitters (e.g., dopamine, histamine) [5,6,7,8]. Increasing evidence links sleep dysfunction with human chronic diseases, including neurodegenerative disorders [9,10,11,12,13,14,15] and disease pathogenesis [16,17,18]. However, the underlying etiology remains poorly understood. This review aims to further elucidate the potential connections between sleep-related genes and diverse human chronic diseases, offering insights into the molecular alterations of these genes and their potential roles in disease diagnosis and treatment.

## 2. Sleep-Related Genes Contribute to Multiple Biological Processes

**Figure 1:**
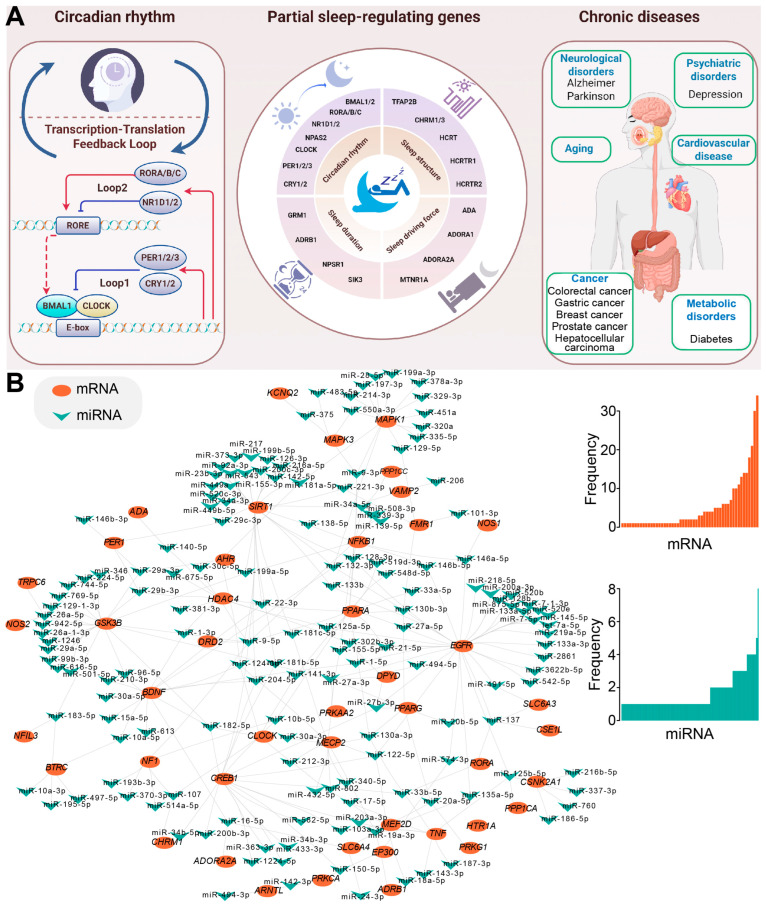
The schema of sleep-related genes and human diseases. (**A**) A schematic overview of sleep-related genes and their association with human diseases created using Figdraw. The left panel depicts the basic circadian rhythm, the middle panel highlights key genes involved in sleep regulation, and the right panel illustrates how dysregulated sleep-related genes and sleep disorders may contribute to a range of chronic human diseases. (**B**) An example illustrating a sleep-related miRNA-mRNA regulatory network. Experimentally validated miRNA–mRNA interactions are sourced from the starBase database [20], and the network is constructed by Cytoscape (3.6.1) [21]. The detailed distributions of interaction numbers are also presented on the right, indicating the complex interactions in miRNA-mediated regulatory networks.

Based on the definitions, characteristics, and functions of sleep-related genes, these genes can be categorized into circadian clock genes, sleep structure regulation genes, sleep drive regulation genes, and sleep duration regulation genes (Figure 1A). For instance, the *PER2* gene influences the biological clock’s oscillation cycle by regulating the transcriptional activation of *BMAL1* [19]. Mutations in *PER2* can lead to advanced sleep phase syndrome, resulting in significantly early sleep and wake times. Numerous non-coding RNAs (ncRNAs), including the widely studied microRNAs (miRNAs), regulate sleep by targeting sleep-related mRNAs within miRNA-mediated regulatory networks (Figure 1B). The complex miRNA–mRNA interactions play a pivotal role in regulating sleep-related processes via dynamically modulating gene expression, and dysregulation of regulatory networks may be linked to sleep disorders, highlighting their importance in maintaining sleep quality and neural health.

The evolutionarily conserved miRNA, miR-137, targets the neuropeptide hypocretin/orexin (*HCRT*), thereby modulating the wake–sleep ratio [22]. Dysregulated miRNAs are implicated in the pathophysiology of central hypersomnias [23]. Specifically, a study found significant changes in the plasma levels of four miRNAs in patients with central hypersomnias: miR-30c, let-7f, and miR-26a were upregulated, while miR-130a was downregulated. The dysregulation of miRNAs may influence the disease’s progression by regulating neuronal differentiation and REM sleep. Another highly conserved miRNA, let-7, targets *Cry2* mRNA, influencing the stability and translation of *Cry2* [24]. miR-132 plays a critical role in the regulation of the *PER* gene, a key component of the circadian rhythm [25]. Light stimulation in the suprachiasmatic nucleus (SCN) induces the transcription of miR-132 via activation of the MAPK/ERK and CREB signaling pathways. miR-132, acting as a negative regulator, inhibits light-induced *PER* gene expression, thereby attenuating the light’s reset effect on circadian rhythms. The potential links between these sleep-related genes and factors like sleep duration and periodicity are increasingly studied based on their distinct functions. Critical sleep-related genes, particularly those involved in circadian rhythms and core regulatory pathways, have been summarized according to their effects on sleep.

### 2.1. Circadian Rhythm-Related Genes Are Associated with Sleep

A number of key genes involved in regulating the circadian rhythm have garnered considerable attention due to their critical roles in the sleep–wake cycle, with their regulatory mechanisms exhibiting hierarchical network characteristics. Studies have revealed significant abnormalities in the circadian rhythm of peripheral clock genes (such as *Per2*, *Bmal1*, and *Nr1d1*) in patients with idiopathic REM sleep behavior disorder (RBD) [26]. The melatonin curve in patients with RBD was delayed by 2 h compared to healthy controls, and their sleep stages were delayed by 1 h. These findings highlight the close association between circadian rhythm-related genes and sleep. For instance, the *BMAL1* gene forms a heterodimer with the *CLOCK* gene, which binds to the E-box sequence, thereby activating the transcription of downstream circadian genes such as *Per1*, *Per2*, *Cry1*, and *Cry2* [27]. Furthermore, RORE—the binding site for ROR and REV-ERB proteins—plays a pivotal role in the regulation of *BMAL1* transcription. ROR activates *BMAL1* transcription via RORE, while REV-ERB inhibits this process, thereby establishing a stable feedback loop that maintains the molecular clock cycle [28]. In *BMAL1*-deficient mice, both sleep and activity cycles were disordered, with no distinct circadian rhythm under constant darkness. These mice exhibited an approximately 6.3% increase in total sleep time, sleep fragmentation, and other disruptions [27]. *BMAL1* expression in skeletal muscle is essential for regulating total sleep, influencing not only sleep volume but potentially affecting the brain’s sleep regulation mechanisms by releasing certain factors [29]. Moreover, in a mouse model with a C-terminal truncated *BMAL1* gene, homozygous mutant mice displayed irregular mRNA and protein expression in the suprachiasmatic nucleus and liver, indicating disruption of the molecular clock mechanism [30]. The C-terminal region of *BMAL1* is pivotal for maintaining biological rhythms and regulating physiological functions. Additionally, sleep deprivation significantly reduced the expression of *CLOCK* and *BMAL1* genes while increasing the expression of the *PER1* gene [31].

The Transcription–Translation Feedback Loop (TTFL) is a fundamental component of the cellular circadian clockwork. Positive regulatory molecules, such as *BMAL1*, *CLOCK*, and *NPAS2*, form heterodimers and bind to the E-box elements of target genes, thereby activating the transcription of *PER* and *CRY*. In contrast, negative regulatory molecules *PER* and *CRY* inhibit the transcriptional activity of *BMAL1*, *CLOCK*, and *NPAS2*, creating a negative feedback loop [32,33] (Figure 1A). A positive correlation can be detected between the expression of *PER1* and total sleep time, as well as NREM sleep duration, while a negative correlation was detected with sleep latency and the α, β, and δ waves in EEG [31]. The *PER2* gene mutation, specifically phosphorylation at the S662 site, is associated with familial advanced sleep phase syndrome (FASPS). The hPER2 S662G mutation linked to FASPS alters the phosphorylation level of PER2 in vitro through tyrosine kinase I (CKI), thereby impacting its circadian clock regulatory function [19]. Additionally, the *PER3* VNTR polymorphism (4 or 5 repeats) is associated with variations in sleep duration and responses to sleep deprivation. In *Per3* (5/5) mice with 5 repeats, EEG power loss after sleep deprivation could be fully compensated, whereas *Per3* (4/4) mice with 4 repeats failed to compensate fully [34]. The *CRY1* gene primarily functions as an inhibitory component in the CLOCK-BMAL1-mediated transcription–translation negative feedback loop. Mutant *CRY1* mice exhibit a significantly prolonged free-running cycle of 28 h, indicating disruption to the stability of their biological clock [35]. *CRY1*-deficient mice display an accelerated free activity cycle, while *CRY2*-deficient mice exhibit a delayed activity cycle [36]. When both *CRY1* and *CRY2* are absent, mice completely lose their free activity rhythm, leading to a disordered biological clock. FASPS is linked to mutations in the *PER2* gene, while the *CRY1* gene is associated with familial delayed sleep phase syndrome (FDSPS) [37].

### 2.2. Other Sleep-Related Regulatory Genes

Due to the unique structure of human sleep, several genes play specific roles in determining the proportions and characteristics of different sleep stages, including non-rapid eye movement (NREM) and rapid eye movement (REM) sleep. For instance, GAD67-positive GABAergic neurons in the ventral tegmental area (VTA) are considered crucial for regulating NREM sleep. These neurons release GABA to inhibit arousal-promoting neurons (e.g., neurons containing appetite neuropeptides), thereby facilitating NREM sleep [38]. Chemical genetic activation of GAD67+ neurons in the VTA significantly increases NREM sleep duration and slow-wave activity, while optogenetic inhibition of these neurons causes rapid awakening from NREM sleep without affecting REM sleep [38]. The *Tfap2b* gene, an upstream regulator, influences the function of GABAergic neurons by affecting the expression of genes related to glutamate decarboxylase (*GAD1* and *GAD2*) and the GABA transporter (*Vgat*) [39]. The *NALCN* gene significantly impacts REM sleep duration; its missense mutation (*Nalcn* Drl) results in a substantial reduction in REM sleep time in mice [40]. Two important muscarinic acetylcholine receptors, *Chrm1* and *Chrm3*, play pivotal roles in the structure of human REM and NREM sleep. Double knockout of *Chrm1* and *Chrm3* leads to a dramatic reduction in REM sleep, nearly eliminating it [41]. Orexin-A, another critical sleep regulator, inhibits both REM and NREM duration. Produced in the hypothalamus, orexin-A promotes wakefulness while inhibiting NREM and REM sleep through two receptors, OX1R and OX2R. Notably, OX2R plays a primary role in mediating the promotion of wakefulness and inhibition of NREM sleep [42]. In OX2R knockout mice, the effects of orexin-A on wakefulness and NREM sleep were significantly attenuated. OX1R also contributed to maintaining wakefulness, though to a lesser extent than OX2R [42]. The orexin system also sustains wakefulness by regulating factors such as histaminergic neuron activity in the tuberomammillary nucleus (TM) and subsequently regulating the transition from wakefulness to NREM sleep through OX2R [43].

Several genes have been implicated in the regulation of sleep-driving forces, the maintenance of sleep or wakefulness homeostasis, and other related functions as demonstrated through various experimental models—including sleep deprivation and physical stimulation. *NPAS2* plays a pivotal role in sleep homeostasis regulation. Under baseline conditions, *NPAS2*-deficient mice exhibited a 41 min reduction in NREM sleep (NREMS) compared to wild-type mice [44]. Furthermore, *NPAS2* deficiency led to more pronounced reductions in NREMS and increased arousal following acute and chronic fentanyl administration [45]. These findings underscore the potential role of *NPAS2* in regulating drug-induced alterations in sleep and wakefulness. Adenosine, a key sleep-promoting substance, acts as an endogenous sleep inducer. Within the A1 receptor/BF pathway, adenosine functions as an inhibitory neuromodulator, suppressing cholinergic neurons in the basal forebrain (BF) via activation of the A1 receptor [46]. Additionally, adenosine inhibits histaminergic neuron activity by activating *ADORA1* receptors [47]. Another adenosine receptor, *ADORA2A*, also influences sleep regulation. Two distinct types of sleep neurons—Type-1 and Type-2—reside in the ventrolateral preoptic nucleus (VLPO). Type-1 neurons exhibit inhibitory responses to adenosine, whereas Type-2 neurons are excited by 5-hydroxytryptamine and adenosine. Notably, Type-2 neurons are activated by the A2A adenosine receptors, thereby promoting sleep [48]. Melatonin, another key sleep regulator, acts as a sleep driver. Upon binding to the MT1 receptor, melatonin activates the associated G protein, resulting in the dissociation of the Gα subunit and the Gβγ subunit. The released Gβγ subunit subsequently activates the BK potassium channel which inhibits neurotransmitter release, thus promoting sleep [49].

Mutations in certain sleep-related genes can significantly impact the overall sleep duration of individuals. These mutations not only affect total sleep time but can also alter sleep and wake cycles. For instance, the *DEC2* gene mutation (hDEC2-P385R) is associated with a short sleep phenotype, causing affected individuals to sleep considerably less than the average population [50]. Similarly, mutations in the glutamate receptor *GRM1* gene, specifically GRM1b-R889W and GRM1-S458A, have been linked to natural short sleep [51]. Individuals with the rare A187V mutation in the *ADRB1* gene typically sleep only 4–6 h per night yet report feeling well-rested [52]. Using optogenetics and calcium imaging, researchers confirmed that *ADRB1*-positive neurons are active during REM sleep and wakefulness, with mutations enhancing neuronal activity, thereby promoting wakefulness [52]. The Y206H mutation in the *NPSR1* gene is also associated with a natural short sleep phenotype. When NPS binds to *NPSR1* it activates downstream signaling pathways, leading to increased phosphorylation of cAMP response element-binding protein (CREB) [53]. This effect was particularly pronounced in *NPSR1*-Y206H mutant mice, where the mutation heightened receptor sensitivity to NPS, thereby promoting wakefulness and reducing sleep duration [53]. Additionally, *SIK3*, which plays a role in intracellular signaling, regulates sleep needs and self-adjusts daily sleep volume, further influencing overall sleep duration [40].

### 2.3. Sleep-Related Genes Are Associated with Multiple Biological Processes

Individuals with poor sleep quality often experience reduced immunity, making them more susceptible to illness. A number of sleep-related genes play a significant role in modulating immune function. For example, activation of *ADORA2A* can increase intracellular cAMP levels, which inhibits the effector function and anti-tumor activity of T cells [54]. Deletion of *ADORA2A* using CRISPR/Cas9 has been shown to enhance the function of CAR T cells, with *ADORA2A*-deficient CAR T cells demonstrating improved tolerance in mice. *ADORA2A* also plays a critical role in tumor immune escape; elevated levels of adenosine in the tumor microenvironment activate A2A receptors via the CD39/CD73 axis, promoting immune evasion [55]. In tumor models, combining *ADORA2A* receptor inhibition with anti-PD-1 therapy significantly improves therapeutic outcomes [55]. Additionally, *ADRB1* influences T cell exhaustion by inhibiting cytokine production and proliferation through the activation of adenylate cyclase and an increase in intracellular cAMP levels [56]. The inhibitory neurotransmitter GABA, involved in sleep regulation, also impacts the immune system, particularly the maturation and inflammatory response of macrophages. GABA enhances the succinate-flavin adenine dinucleotide (FAD)-lysine-specific demethylase 1 (LSD1) signaling pathway [57]. Additionally, 5-HT modulates sleep by influencing the polarization of human macrophages through various receptors, including 5HT2B and 5HT7, which play a critical role in regulating inflammation [58]. Specifically, 5-HT alters the gene expression profile of macrophages via activation of 5HT2B and 5HT7 receptors, promoting the expression of anti-inflammatory genes while inhibiting the expression of pro-inflammatory genes.

## 3. Dysregulated Sleep-Related Genes Have a Critical Role in Human Diseases

### 3.1. Sleep-Related Genes May Be Associated with Metabolism-Related Diseases

The fast-paced nature of modern life significantly impacts sleep, with sleep deprivation being closely linked to increased stress. Insufficient sleep disrupts eating patterns, leading individuals to reduce main meal intake while increasing snacking and high-calorie food consumption, particularly during the evening [59,60]. Interestingly, although extended wakefulness due to sleep deprivation theoretically increases energy expenditure, it is insufficient to offset the additional calories consumed, potentially due to a reduction in leptin levels which normally suppress appetite [59,61]. Circadian clock genes play a key role in regulating metabolism. For instance, *PER2* influences lipid metabolism by inhibiting PPARγ, and the loss of *PER2* leads to increased PPARγ activity, resulting in metabolic disruptions [62]. Another key circadian gene, *CRY1*, also impacts metabolic processes. *CRY1* mutant mice exhibit polyuria and excessive thirst at 20 weeks of age, suggesting a potential connection to diabetes [35].

Orexin regulates both sleep architecture and wakefulness, while also serving as a critical metabolic modulator. In high-fat diet-induced obese mice, selective inactivation of Ox1R reduced glucose utilization in brown adipose tissue and skeletal muscle, ultimately impairing insulin sensitivity [63]. This highlights the dual role of the orexin system in glucose homeostasis: Ox1R activation typically enhances insulin sensitivity and promotes BAT thermogenesis, whereas Ox2R activation is linked to diminished glucose tolerance and insulin resistance [63]. Furthermore, circadian clock gene disruptions not only precipitate insulin resistance, diabetes, and obesity, but also exacerbate the risk of kidney stone disease through oxidative stress and inflammation [64]. For instance, insulin resistance can lead to increased urine acidification and calcium excretion, while diabetes induces oxidative damage to renal tubular cells, thereby promoting stone formation [64]. Additionally, insufficient sleep or circadian rhythm disruptions often manifest as hormonal imbalances, resulting in symptoms such as skin discoloration and emotional instability. Specific circadian genes regulate hormone secretion, as evidenced by *PER2*-deficient mice, which lack normal cortisol rhythms during the light–dark cycle [65]. In these mice, cortisol levels significantly increased after mild restraint stress and remained elevated long-term, indicating a dysfunction in the hypothalamus–pituitary–adrenal (HPA) axis [65]. The liver’s circadian clock can regulate choline kinase (*CHK*) gene expression via the BMAL1-REV-ERBα axis, influencing lipid metabolism [66]. REV-ERBα exhibits a clear circadian rhythm by inhibiting *CHK* expression, thus reducing phosphatidylcholine synthesis. Disruption of this rhythm in the absence of *BMAL1* leads to elevated phosphatidylcholine levels, resulting in altered lipoprotein profiles, an increase in non-HDL cholesterol, and the development of conditions such as fatty liver, dyslipidemia, and metabolic syndrome. Additionally, REV-ERBα regulates transcription by directly binding to the RORE sequence in the *CHK* gene promoter [66].

### 3.2. Sleep-Related Genes May Be Associated with Cardiovascular Diseases

The aging population, dietary changes, and other societal factors have increasingly highlighted cardiovascular health concerns. Sleep and circadian rhythms are critical in regulating cardiovascular functions. Healthy sleep patterns significantly reduce the risk of cardiovascular disease (CVD), coronary heart disease (CHD), and stroke [67]. *PER2*-deficient mice exhibit larger myocardial infarct sizes and reduced lactate production during cardiac ischemia, potentially impairing the heart’s energy supply. As a circadian gene, *PER2* may offer a protective role in the cardiovascular system. Treatment with TNF-α or H_2_O_2_ disrupts mitochondrial membrane potential, and *PER2* knockdown exacerbates this damage [68]. The BK channel, another factor promoting sleep, also plays a protective role in cardiovascular health by enhancing Ang II’s inhibitory effect on BK channels [69]. Pharmacological activation of BK channels or their associated genes can safeguard cardiac function in diabetic mice and mitigate myocardial ischemia/reperfusion injury [69]. Moreover, circadian rhythms regulate the sympathetic–parasympathetic balance of the heart, with *PER3* gene polymorphisms influencing autonomic nervous system (ANS) activity [70]. Sleep deprivation leads to reduced heart rate variability and heightened sympathetic nerve activity. Other circadian genes, such as *CRY1*, have been linked to hypertension risk, with serum levels serving as diagnostic markers for individuals with abnormal anthropometric indicators [71].

As a key regulator of sleep, 5-HT receptors also play a role in maintaining normal cardiac function. For instance, the absence of 5-HT(2B) receptors results in cardiomyocyte dysfunction [72]. 5-HT(2B) receptor-deficient mice exhibit left ventricular dilation and impaired systolic function indicative of myocardial tissue loss and disrupted muscle fiber alignment [72]. The 5-HT(2A) receptor mediates cardiac cell hypertrophic responses to 5-HT and is associated with *HDAC4* phosphorylation [73]. Selective 5-HT2A receptor antagonists significantly reduce the phosphorylation of CaMKII and *HDAC4* in mice with aortic coarctation, thereby inhibiting cardiac hypertrophy [73].

### 3.3. Sleep-Related Genes Are Associated with Cancer

Insomniacs face a 24% higher cancer risk compared to the general population, likely due to factors such as chronic inflammation, impaired immune function, and hormonal alterations caused by sleep deprivation [74]. One sleep-related gene, the circadian clock gene *BMAL1*, is involved in cell proliferation and apoptosis [75]. Circadian clock genes are crucial for regulating the cell cycle, linking them closely to cancer development. Loss of *Per1* significantly impacts the proliferation and apoptosis of oral squamous cell carcinoma (OSCC) cells. Cells overexpressing *Per1* show a marked reduction in proliferation and a substantial increase in apoptosis [76]. The loss of *PER1* may influence OSCC progression via the AKT/mTOR signaling pathway, while *PER1* modulates cell proliferation through glycolysis regulation and the PI3K/AKT pathway, positioning it as a tumor suppressor [77]. In trastuzumab-resistant gastric cancer cells, *PER1* silencing disrupts the circadian rhythm of the PER1-HK2 axis, potentially reversing drug resistance [78]. Specifically, *PER1* interacts with PPARγ to enhance its transcriptional activity, promoting *HK2* expression, altering glucose metabolism, and impacting the proliferation and survival of cancer cells. This suggests that targeting *PER1* could be a promising strategy for overcoming trastuzumab resistance in gastric cancer [78]. Moreover, *PER1* may modulate immune pathways through NK cell clocks [79], and its role in regulating the cell cycle and DNA damage repair underscores its potential as a tumor suppressor [80,81], making it a valuable target for cancer treatment [82,83,84,85]. Similarly, the circadian clock gene *PER2* is closely linked to tumor suppression. Mice deficient in *PER2* exhibit heightened cancer susceptibility, particularly following radiation exposure [86]. Lymphocytes from mPer2 mutant mice display resistance to apoptosis after radiation, leading to the accumulation of damaged cells. As a tumor suppressor, *PER2* exerts significant inhibitory effects on breast cancer cells. Overexpression of *PER2* in the MCF-7 breast cancer cell line leads to a notable increase in the proportion of cells in the G1 phase and a decrease in the S phase. This effect is more pronounced when *PER2* and *Cry2* are co-expressed [87]. Additionally, *PER2* expression induces apoptosis in MCF-7 cells. As a key regulator of the circadian cycle and transcriptional control of other clock genes, *PER2* modulates various physiological systems, including metabolism, sleep, immune response, cardiovascular health, and renal function, highlighting its complex role in sleep disorders and their assessment and treatment [88]. *BMAL1* also contributes to colorectal cancer metastasis by stimulating exosome secretion, providing insight into the role of circadian rhythms in cancer progression [89]. *BMAL1* depletion enhances the sensitivity of adrenocortical carcinoma cells to DNA damage-based therapies both in vitro and in vivo, implicating *BMAL1* as a potential target in cancer therapeutics [90]. Its influence on cancer cell behavior suggests that *BMAL1* may be a promising therapeutic target in colorectal cancer [91]. Moreover, *BMAL1* regulates the stemness and tumorigenesis of gliomas via the Wnt/β-catenin signaling pathway, with its interaction with glioma stem cells significantly impacting glioma initiation and progression [92].

*NPAS2*, a gene that promotes sleep recovery, also acts as a circadian regulator. In prostate cancer, it enhances cell survival by promoting glycolysis and inhibiting oxidative phosphorylation. Overexpression of *NPAS2* increases glucose uptake and lactate production, thereby improving cellular glycolytic capacity [93]. In hepatocellular carcinoma, *NPAS2* promotes cell survival and proliferation by upregulating phosphatase *CDC25A* expression through direct binding to the E-box element in the *CDC25A* promoter [94]. This leads to the dephosphorylation of *CDK2*, *CDK4*, and *CDK6*, activating these kinases and promoting cell cycle progression [94]. Another sleep regulator, *SIK3*, promotes cancer cell proliferation through the mTOR signaling pathway [95]. Mutations in the *GRM1* gene, which is involved in regulating sleep duration, are associated with decreased sleep duration, as seen with specific mutations such as GRM1b-R889W and GRM1-S458A linked to natural short sleep. In mouse models, inducible RNA interference-mediated inhibition of *GRM1* expression results in reduced cell proliferation, increased apoptosis, and suppression of downstream MAPK and PI3K/AKT pathways [96]. Inhibition of *GRM1* is associated with increased apoptosis markers and reduced proliferation markers such as Ki-67. Additionally, *GAD1*, which regulates human sleep architecture by synthesizing the GABA-inhibitory neurotransmitters, facilitates tumor cell proliferation through GABA-mediated β-catenin activation. This occurs via the inhibition of glycogen synthase kinase 3β through GABA_B receptor activation, enhancing β-catenin signaling [97]. In a mouse model, knockout of *GAD1* significantly slowed tumor growth. GABA induced a non-T cell inflammatory tumor microenvironment by inhibiting the activity and infiltration of CD8+ T cells, and it also suppressed the production of chemotactic factors, such as *CCL4* and *CCL5*, which typically recruit T cells and dendritic cells to the tumor site [97]. This highlights GABA’s role in immune evasion. As the connection between sleep genes and cancer becomes clearer, researchers have begun exploring chronotherapy as a potential treatment approach. Circadian rhythm genes offer an optimized framework for improving therapeutic outcomes. Since different cell types exhibit varying drug sensitivities, administering certain chemotherapy drugs at specific times of day can reduce toxicity and enhance efficacy [98,99].

**Figure 2:**
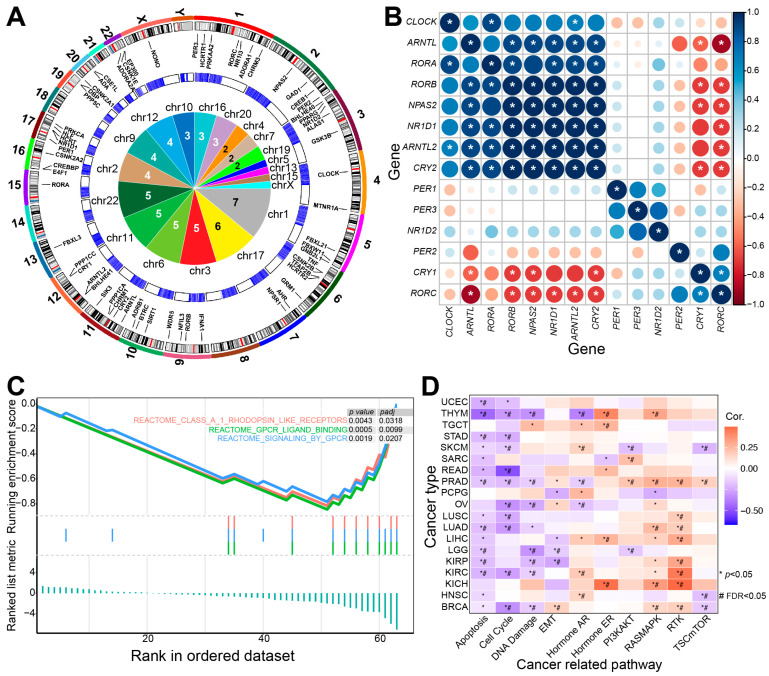
The potential role of sleep-related genes in tumorigenesis. (**A**) Gene distributions of sleep-related genes on human chromosomes. The outer circle shows the distributions of core circadian rhythm-related genes on human chromosomes. The middle circle displays the expression distributions in normal brain tissues (data obtained from The Cancer Genome Atlas (TCGA) using “TCGAbiolinks” [100]) and the inner panel provides detailed pie distributions of genes on human chromosomes. Chromosomes 1 and 17 contain more genes than other chromosomes. (**B**) Expression correlations of core sleep-related genes based on the glioblastoma multiforme (GBM) dataset from TCGA (TCGA-GBM). * indicates a significant Spearman correlation (*p* < 0.05). (**C**) Gene set enrichment analysis (GSEA) reveals that these sleep-related genes are significantly enriched in specific biological pathways. (**D**) These genes may contribute to tumorigenesis by interacting with specific pathways (analyzed using the GSCA platform [101]).

These sleep-related genes have garnered significant attention in cancer research due to the intricate relationship between sleep and tumorigenesis. They are distributed on most human chromosomes, especially on Chromosomes 1 and 17 (Figure 2A). Positive or negative expression relationships can be detected between some sleep-related genes (Figure 2B), and some genes play a role in cancer-related pathways, such as GPCR-related signaling pathways, apoptosis, the cell cycle, DNA damage, and RTK pathways, indicating the potential contributions to tumorigenesis (Figure 2). Dysregulation of gene expression in specific cancers indicates the potential involvement in both physiological and pathological processes, indicating that gaining insights into the mechanisms of sleep regulation could facilitate the development of cancer therapies.

### 3.4. Sleep and Mental Health

As the central regulator of sleep, the nervous system is profoundly impacted by sleep loss or other sleep disorders. Common neurological diseases, such as Alzheimer’s disease, Parkinson’s disease, and schizophrenia are often closely linked to sleep disturbances. Many patients with neurological conditions experience varying degrees of sleep difficulties, including sleep fragmentation. Variations or functional alterations in genes that regulate sleep increase the risk of these neurological disorders. Patients with schizophrenia, for example, typically exhibit poor sleep quality, with disrupted sleep structure characterized by reduced deep sleep and shortened REM latency [102]. Dysregulation of the circadian rhythm genes *CRY1* and *PER2* is commonly observed in these patients. In individuals with first-episode schizophrenia, the mRNA expression of *CLOCK*, *PER2*, and *CRY1* genes significantly decreases in the early stages, suggesting that the circadian rhythm disturbance may be part of the pathological process of schizophrenia rather than a result of long-term drug treatment [103]. In contrast to the worsening effects of sleep gene loss on neurodegenerative diseases, experimental mice lacking *BMAL1* demonstrated improved performance. *BMAL1* deficiency inhibited the aggregation of tau protein and α-synuclein (aSyn), as well as related pathological changes [104]. Astrocytes lacking *BMAL1* showed enhanced autophagy and phagocytosis, likely due to increased *BAG3* expression. Overexpression of *BAG3* significantly reduced the spread of α-synuclein. *BAG3* was found to be highly expressed in the astrocytes of patients with Alzheimer’s disease, suggesting a potential therapeutic target for astrocyte-specific interventions in neurodegenerative diseases [104]. Additionally, the complex between the 5-HT2A receptor and mGluR2 receptor has been extensively studied in relation to schizophrenia. These receptors can form functional complexes, integrating serotonin and glutamate signaling to regulate G-protein coupling patterns [105]. Activation of mGluR2 can inhibit behavioral responses, such as head torsion induced by 5-HT2A receptor agonists. This receptor complex plays a key role in regulating hallucination-related behaviors and may represent a promising therapeutic target for schizophrenia [105].

Depression remains a major global health burden, with many antidepressants facing challenges related to poor efficacy or side effects. Sleep-related genes may offer promising new therapeutic targets. Up-regulation of *ADORA2A* (A2AR), a receptor for adenosine, has been linked to the onset of depressive symptoms, and *ADORA2A* antagonists are considered potential antidepressants [106]. A2AR activation increases the firing rate of A2AR-positive neurons in the lateral ventricle, inhibiting the activity of neighboring neurons. This activation can induce depression-like behavior while inhibiting A2AR-positive neurons reduces these behaviors [106]. Stress-induced up-regulation of *ADORA2A* can lead to alterations in neuronal function, promoting depression-like symptoms. Therefore, *ADORA2A* up-regulation may serve as both a biomarker and a therapeutic target for depression. In addition, GABA, an inhibitory neurotransmitter, plays a pivotal role in sleep regulation and significantly affects appetite and mood. A long-term high-fat diet can reduce the responsiveness of AgRP neurons to hunger, anxiety, and depression stimuli [107]. This decreased sensitivity weakens the GABAergic output of AgRP neurons to downstream MC4R dBNST neurons, contributing to severe psychological disorders. Enhancing GABA_A_ R-α5 signaling or inhibiting 5-HT3 receptor signaling can significantly alleviate anxiety and depression induced by a high-fat diet, while also inhibiting excessive food intake and weight gain [107]. Furthermore, the ERK MAPK signaling pathway is vital in regulating sleep and plays a key role in learning and memory. Activation of this pathway promotes the expression of genes involved in neuroplasticity, which are essential for stress-induced learning and memory processes [108]. Specifically, ERK MAPK activation leads to the phosphorylation (Ser10) and acetylation (Lys14) of histone H3 through downstream molecules like MSK1 and Elk-1, forming the dual modification H3S10p-K14ac. This modification enhances the transcriptional activation of immediate early genes such as c-fos, which is considered a key marker for memory consolidation.

Brain functions, such as attention and memory, are closely linked to sleep quality and status. Genes involved in regulating sleep or circadian rhythms may also influence other functions in the same or different brain regions, including memory, learning, and social interaction. For instance, deletion of the circadian rhythm gene *BMAL1* results in significant social impairments and repetitive stereotyped behaviors in mice, resembling autism spectrum disorders [109]. *BMAL1* deficiency may also induce dry mouth and eyes through down-regulation of ITPR2/3, suggesting a potential therapeutic avenue for these symptoms [110].

## 4. Conclusions

To explore the molecular alterations of sleep-related genes and their potential associations with human diseases, this study discussed the complex mechanisms of sleep regulation, particularly their roles in chronic human diseases. The relationships between sleep-related genes and various conditions, including neurological, metabolic, cardiovascular diseases, and cancer were further examined, along with the central genes involved. Given the profound connection between sleep and disease, research efforts are increasingly focusing on utilizing genetic and circadian rhythm information to regulate sleep quality. This approach is paving the way for the diagnosis and treatment of related diseases based on insights from human circadian and sleep gene data. The sleep regulation mechanisms will support the development of new therapeutic strategies and pharmacological treatments for diseases associated with sleep and circadian rhythms.
